# Role of Short Term Video Encephalography with Induction by Verbal Suggestion in Diagnosis of Suspected Paroxysmal Nonepileptic Seizure-Like Symptoms

**DOI:** 10.1155/2016/2801369

**Published:** 2016-11-17

**Authors:** Soaham Dilip Desai, Devangi Desai, Trilok Jani

**Affiliations:** ^1^Department of Neurology, Pramukhswami Medical College, Shree Krishna Hospital, Karamsad, Anand, Gujarat 388325, India; ^2^Department of Medicine, Pramukhswami Medical College, Shree Krishna Hospital, Karamsad, Anand, Gujarat 388325, India

## Abstract

*Purpose*. To determine the diagnostic yield and utility of STVEEG with verbal suggestion in diagnosis of patients presenting with transient unresponsiveness and suspected psychogenic nonepileptiform seizures.* Methods*. A retrospective analysis of STVEEG records of patients with transient unresponsiveness and suspected PNES between 1 Jan 2009 and 28 Feb 2014 was done.* Results*. Amongst 155 patients [38 males, 117 females], with mean age 32 [8–67], PNES were identified in 109 [70.3%], focal epilepsy was identified in 24 [15.4%], and actual seizure was recorded in 7 [4.5%]. Nine [5.8%] patients were found to have both epilepsy and PNES. Primary generalized epilepsy was diagnosed in 2 [1.2%]. A diagnosis of other paroxysmal nonepileptiform events [tachyarrhythmia and heart block] was done in 3 [1.9%]. A normal EEG and no inducible episode and hence an uncertain diagnosis at the end of STVEEG were seen in only 17 [10.9%] patients. A STVEEG of approximately one hour duration was able to establish the diagnosis in 138 [89.1%] patients with transient unresponsiveness.* Conclusion*. STVEEG with verbal suggestion is a useful and cost effective diagnostic test for diagnosis of PNES. It can be a good modality for diagnosis in patients with transient abnormalities in sensorium in the outpatient settings in developing countries.

## 1. Introduction

When a patient presents with attacks of transient unresponsiveness, the usual differentials are complex partial seizures and syncope. Paroxysmal nonepileptic seizure-like symptoms (PNES) form another differential in this group of patients. Frequency of PNES varies between 10 and 30% across studies on assessing diagnoses amongst patients presenting with episodes of unresponsiveness [1, 2]. It becomes important to differentiate between complex partial seizures (CPS) and PNES amongst patients presenting with episodes of unresponsiveness. While there are important clinical features which can help in differentiating a CPS and PNES, it is often difficult to elicit the history of important semiology features [1, 2]. If such an episode can be observed and the ictal electroencephalography (EEG) pattern is reviewed, then the differentiation can be done. Long term video electroencephalograph (LTVEEG) is a well-established technique in evaluation of medically refractory epilepsy, for characterization of epilepsy, epilepsy classification, presurgical evaluation, and diagnosis of PNES [3]. As this test is resource and labor intensive, it is not available at all centers. Also the need of admission and cost of the procedure make it difficult for patients to undergo the evaluation, especially in financially underprivileged rural population.

Short term VEEG (STVEEG) is a video EEG test done for a short duration of few hours, often on an outpatient basis, which is helpful in evaluation of patients with frequent intractable events. It has been documented to help in easier diagnosis in patients with high clinical suspicion of having PNES [4]. However, its clinical utility and place in routine clinical practice are still not well defined [4, 5]. Many provocative techniques have been described to induce events during VEEG monitoring when PNES are suspected clinically [1, 2, 6]. Some of these methods like injection of saline or other placebos for induction have been utilized and proposed by many, but these techniques have their own ethical and technical limitations [6]. The technique of simple verbal suggestion for provocation of events without placebo injections has also been described but its utility is still under question [5–7]. Hence, we undertook this study to assess the role of STVEEG with the technique of induction by verbal suggestion (VS) in the diagnosis of PNES in patients presenting with episodes of unresponsiveness in the rural setting. We intended to study the yield of STVEEG with VS in diagnosis of patients with PNES in terms of number of patients in whom PNES could be diagnosed in all patients with clinically suspected PNES. Our secondary objective was to assess the mean timing and range of total duration of STVEEG required for confirming the diagnosis of PNES and to identify whether STVEEG can also identify patients who have both epilepsy and PNES.

## 2. Methodology

A retrospective analysis of records of all patients who underwent short term video electroencephalography (STVEEG) from 1 Jan 2009 to 28 Feb 2014 in the Electroencephalography (EEG) Laboratory of Neurology Department of Shree Krishna Hospital, a rural based medical teaching hospital attached to Pramukhswami Medical College, in Karamsad in Gujarat in Western India was done. The demographic data was available from the electronically captured database in the EEG software. The pretest clinical details, provisional diagnosis prior to EEG analysis, and other clinical data were available from EEG reference form and other medical records of the patient. From these records, details of all patients with suspected paroxysmal nonepileptic seizure-like symptoms (PNES) were separated and clinical information required for the study was collected from the STVEEG reports using a structured format. A diagnosis of epilepsy was done from the clinical details about seizure semiology, EEG findings in the interictal period of recording as well as ictal electrographic rhythm when available. PNES was diagnosed on the basis of clinical semiology accompanied by the associated semiology on video recording as well as EEG finding during the episode. All patients with 2 or more episodes of transient unresponsiveness, with clinical features not definitely suggestive of seizure or syncope, who underwent STVEEG at our EEG lab after informed consent, were included in our study. Patients who on history were likely to have ≥2 different types of episodes (namely, both seizures and PNES) were also included in the study. Patients who on clinical history and examination had a diagnosis of definite seizures or syncope episodes only and not PNES were excluded. The project was approved by the institutional ethics review committee.

During the STVEEG, in all patients with suspected PNES, a standard protocol has been used by us during the entire period of recordings and review. All patients undergo an EEG recording in a quiet room in a reclining position and are instructed to lie down in a comfortable position and try to induce sleep. After a baseline record of 4 minutes, a 3-minute period of hyperventilation (HV) is recorded followed by intermittent photic stimulation (PS) over a range of 2 Hz to 30 Hz. When PNES is suspected, at the time of explanation of need and procedure of EEG testing, a verbal suggestion is given by the neurologist (SD) that “we would be attaching electrodes on your head to record the electrical activity in your brain during this test. You should think and recollect about what had happened during the episodes that you suffer from and you would have the episode like you have been having and this would be recorded on video for us to see and to show you what happens in such episode.” During the EEG recording, after the routine EEG is recorded for 20 minutes, the EEG technician (TJ) would give a verbal suggestion again that the patient would have the episode of seizure just like he/she usually has and the patient is told to recollect the event that usually happens and his/her feelings during the episode and its immediate period after it. When the episode of seizure would occur, the semiology would be recorded based on the video finding and accompanied EEG findings would help in confirming the diagnosis. Typically the STVEEG would be recorded for a period of about 45 minutes to one hour. The STVEEG is started within about half an hour of suggestion given by the neurologist. We strive to do the STVEEG recording early after suggestion, in order to have consecutive “dual” verbal suggestion by different persons in a short time period to improve the diagnostic yield of the study.

A diagnosis of PNES was considered when a patient has normal baseline EEG; a patient has an inducible episode with characteristic semiology as he/she usually has, with clinical semiology suggestive of PNES, with normal concomitant ictal EEG/only movement artifacts during the episode. A diagnosis of CPS was made if there was abnormal baseline EEG with interictal epileptiform discharges and/or a CPS was recorded during the EEG with characteristic ictal EEG. We also reviewed any other paroxysmal events like arrhythmia recorded on associated ECG record. The STVEEG record was considered inconclusive if the baseline EEG was normal and no episode was inducible by verbal suggestion during the period of STVEEG record.

Demographic and clinical information including age, gender, education, occupation, duration of symptoms, comorbid medical history, and medication history were all recorded. The final diagnosis of each patient, namely, epilepsy, PNES, both epilepsy and PNES, and inconclusive STVEEG, was also recorded. The period for which the STVEEG recording was done as well as the time to record a PNES after verbal suggestion is given to each individual patient was also recorded.

All the findings of the study were recorded on a Microsoft Excel sheet format and the percentage of patients with PNES, epilepsy, both epilepsy and PNES, or other diagnoses was calculated. Diagnostic yield of the STVEEG with induction by VS method was done by calculating the number of positive tests divided by the number of total tests performed, in patients with suspected PNES. Evaluation of the mean duration of VEEG record required for inducing an event during the VEEG recording and the range of total duration of recording required was also calculated. The quantification of number of patients in whom diagnosis of PNES was changed to epilepsy or other abnormalities and vice versa was also done. The quantification of number of patients in whom a final diagnosis could not be done and PNES also could not be induced was also done.

## 3. Results

We studied cases of 155 patients (38 males, 117 females), ranging between 8 years and 67 years age (mean 32) who underwent STVEEG with induction by VS. We could diagnose PNES episodes in 109 (70.3%) after doing STVEEG with induction by VS. An episode of PNES could be induced by VS in all of these patients during STVEEG. We could diagnose epilepsy in 26 (16.6%) of patients at the end of STVEEG record in the study population. Amongst these patients, interictal EEG abnormalities consistent with focal epilepsy were identified in 24 (15.4%) and primary generalized epilepsy was diagnosed in 2 (1.2%) patients. An actual seizure was recorded in 7 (4.5%) patients during the STVEEG record. Nine (5.8%) patients were found to have both epilepsy and PNES. A diagnosis of other paroxysmal nonepileptiform events (tachyarrhythmia and heart block) was done in 3 (1.9%). A normal EEG and no inducible episode and hence an uncertain diagnosis at the end of STVEEG were seen in only 17 (10.9%) patients (see Table 1).

The mean STVEEG recording time was 47 (35–75) minutes and a PNES episode could be induced in a mean period of 26 (13–58) minutes after beginning the EEG recording, thus confirming the diagnosis. A STVEEG record of approximately one-hour duration was able to establish the diagnosis in 138 (89.1%) patients with transient unresponsiveness in our study population.

## 4. Discussion

In this study, we intended to assess the utility of STVEEG with induction by VS in confirming the diagnosis in patients with recurrent episodes of unresponsiveness with clinical suspicion of PNES. We found that, in 70 percent of patients with suspected PNES, an episode of PNES could be induced by VS and thus a definite diagnosis of PNES could be confirmed in these patients. Our study suggests that STVEEG with induction by VS has a very high yield in confirming the diagnosis when PNES is suspected. Different studies have reported different yields of VEEG methods in diagnosing PNES [1, 2, 6–8]. Also different groups have used different methods for evaluation. There are different types of studies, those which do not use any induction methods to diagnose PNES and those that use them (Table 2) [3, 7–19]. The studies describing only VEEG without induction have demonstrated lower rates of diagnoses of PNES as compared to studies utilizing induction methods [3, 18]. Different studies have used different methods of induction like hyperventilation, photic stimulation, placebo injection, rubbing alcohol or spirit solution on skin, and colored patch application on neck while telling the patient that this would provoke a seizure and have shown comparable yield in diagnosis of PNES [6–9, 12].

Methods utilizing hyperventilation and photic stimulation have been criticized in literature as methods of induction of PNES citing the reason that reflex epilepsies can be triggered by photic stimulation and absence seizures can be induced by hyperventilation [13, 14]. In our study, all patients underwent hyperventilation and photic stimulation as a part of standard activation procedure as in traditional EEG studies. The induction by verbal suggestion was done by the technologist only after the hyperventilation and photic stimulation were completed. However, these procedures can also contribute to having additional impact on activation of seizures as well as induction of PNES episodes during the recording. Methods of induction by “provocation of seizures” by using saline injections or other placebos can have ethical limitations and the inherent deceit in this approach may negatively affect the physician-patient relationship and impede future treatment efforts [13, 14]. Also some patient may not consent for receiving “injections or patch applications to induce seizures.” We feel that our method of dual verbal suggestion to the patient to recollect the chain of events preceding the episodes in mind and this leading to the induction of episode avoids the phenomenon of deceit involved in provocation methods using saline injections or placebo applications. Also our method allows us to maintain a good relationship with the patients, empathize with the patients, and give them the confidence that we have observed the event, understood it, and would be able to help them improve their health in a better way. Also it also prevents the rare phenomenon of occurrence of reflex seizures associated with techniques like HV and PS. Limited studies using induction by VS have shown good yield of diagnosing PNES [11, 12, 17]. In our study, we used similar technique of verbal suggestion but modified it in a way that the patients receive the suggestion twice; first the neurologist suggests that the patients will have an episode during the EEG recording while informing the patient about the need to do VEEG and then the EEG technician further enhances the verbal suggestion during the recording by suggesting the same verbally again. We found that our study using this technique of VS yielded an even higher detection of PNES in our study compared to other studies using VS [11, 12, 17]. However, as the studied patient population is different across various studies, there is a lot of heterogeneity and direct comparison between various studies is not correct. While most of the studies describe only the yield in terms of method of VEEG (i.e., LTVEEG or STVEEG), there is no significant study comparing different provocative methods/suggestion techniques and their sensitivity in precipitating and diagnosing a PNES in the same patients.

We also found that we could diagnose patients with epilepsy as well as other disorders like cardiac arrhythmias mimicking seizures in our study population. Amongst all the patients suspected to have PNES clinically, we found that nearly twenty percent of patients had epilepsy or cardiac arrhythmia as the diagnosis at the end of STVEEG. Thus, STVEEG can also help in diagnosing other disorders in patients clinically suspected to have PNES. A suggested limitation of the technique of induction by VS is that the episode induced may not be the usual episode that the patient suffers from and may lead to misdiagnosis of a seizure episode as a PNES. However, this can be taken care of by confirming with the patients' relatives and event witnesses whether the episode observed during the STVEEG is similar to the episode the patient usually has and in cases of doubt the duration of VEEG can be increased or a LTVEEG can be planned. Our method of verbal suggestion (where we suggest the patient to recollect the old events and that they would have an episode like they always have) leads to precipitation of the usual event that the patient is having (if they are indeed PNES events) and does not lead to completely different events which could occur with provocative methods involving placebo injections or applications. At the same time, patients who are not having PNES (epilepsy or other physiologic nonepileptic events) would report that they did not have any event during the record like they usually have despite thinking about the event. A review of the interictal EEG record of the patient can help in identifying any interictal epileptiform discharges which would suggest epilepsy. In our study, we could identify interictal epileptiform activity in nearly fifteen percent of our patients and diagnose epilepsy in these patients. In our study, we could also diagnose patients who had 2 different types of episodes together (epilepsy with coexistent PNES). Nearly six percent of our subjects had both coexistent epilepsy and PNES. Some of the patients with suspected PNES in clinical practice are found to completely deny remembering anything and would not have any event inducible during the recording. In our study also, no event could be recorded and EEG was normal in nearly eleven percent of the patients making the cause of episodes undetermined in that STVEEG test.

While LTVEEG is the gold standard method to diagnose epilepsy type, localize ictal onset region (for presurgical evaluation), and rule out associated PNES, its use is often limited by multiple reasons including inpatient stay, procedure cost, and labor intensiveness. When clinical suspicion of PNES is higher, doing a LTVEEG study may not be very cost effective compared to STVEEG. In our study, we found that, in patients with recurrent episodes and suspected PNES, a STVEEG of duration of nearly one hour with induction by VS could provide a diagnosis in approximately ninety percent of all studied population. Strength of our study is that, throughout our study, we have used a consistent standardized method of induction by VS of PNES in patients in the entire period of the study. Some of the studies on the subject have only described the yield of the procedure of STVEEG and have used varied methods of provocation of PNES across different patients in each individual study [7, 11, 16, 17].

The fact that our study is a retrospective study done at a single center may be considered to be one of the limitations of our study. However, the fact that all the patients were evaluated by single team of neurologist and EEG technician using a standardized method of evaluation suggest that the findings observed are indeed de facto. In this study we had only included patients with history of 2 or more episodes of unresponsiveness. We had not included patients presenting with subjective focal motor or sensory or subjective sensations with preserved responsiveness as this could also occur with focal seizures and could be accompanied by a normal surface EEG. We would also like to remind the readers that seizures do not always produce surface EEG changes and that some patients with seizures may exhibit nonepileptic behavior in response to their unpleasant and or frightening seizure experiences. Hence, though STVEEG with induction by VS is a cost effective method for diagnosis and documentation of PNES, it should be done with extreme caution, by clinicians with expertise in epilepsy as well as PNES.

## 5. Conclusions

STVEEG would be a very cost effective method of diagnosing PNES and differentiating between epilepsy and PNES. In comparison to a routine simple EEG which can neither rule out epilepsy nor confirm PNES, STVEEG with verbal suggestion can confirm PNES and reasonably rule out epilepsy. Our method of induction by VS without using any “provocation” techniques also does not involve the phenomenon of deceit and does not jeopardize the doctor-patient relationship. If it can be effective in diagnosing PNES, it can decrease the need of long term VEEG which is very costly and laborious. If its value and utility in terms of diagnosing and confirming PNES, in terms of its sensitivity, can be evaluated using multicentered studies, it can be utilized by all centers, especially in developing or underdeveloped countries to reduce the treatment and evaluation costs of seizures or seizure-like events.

## Figures and Tables

**Table 1:** Details of findings on STVEEG.

	Male	Female	Total
Recording	38	117	155
PNES	19	90	109 (70.3%)
Epilepsy	6	20	26
Focal epileptiform discharges	5	19	24 (15.4%)
Primary generalized discharges	1	1	2 (1.2%)
Actual seizure recorded	3	4	7 (4.5%)
Others (arrhythmia)^*∗*^	2	1	3 (1.9%)
Undetermined (normal EEG and no episode inducible)	6	11	17 (10.9%)
Both epilepsy and PNES	2	7	9 (5.8%)

^*∗*^1  patient had ventricular tachycardia and 2 patients had heart block.

PNES: paroxysmal nonepileptic seizure-like symptom.

EEG: electroencephalography.

STVEEG: short term video EEG.

**Table 2:** Comparison of findings of our study with those of different other studies, in different settings, using different methods showing diagnostic yield of study in diagnosing epilepsy as well as PNES.

Reference	*N*	Pretest diagnosis	Mean age (range)	Method	Mean recording time (min⁡)	Epilepsy %	PNES %
Our study (2014)	155	PNES likely	32 (8–67)	STVEEG + VS (Double)	47 (35–75)	16.6	70.3

Seneviratne et al. (2012) [17]	175	E: 77.7% P: 22.3%	36 (16–87)	OVE + VS + HV + PS	230	17.3	37.1

McGonigal et al. (2002) [16]	143	“attacks”	34 (14–75)	STVEEG + VS + HV + PS	40–50	4.9	35.7

Varela et al. (2007) [7]	52	PNES	Ns	OVEM + VS + HV + PS	Ns	0	67

Ghougassian et al. (2004) [3]	131	E: 55.7% P: 6.9% NC: 37.4	44.5 (16–88)	LTVEEG	1–13 days	43.5	24

Benbadis et al. (2004) [10]	74	PNES	Ns >18	STVEEG + VS + HV + PS	120	0	63.5

Rowan et al. (1987) [18]	166	Ns	40.5 (16–88)	Daytime No VS	360–480	48	43

Angus-Leppan (2007) [19]	1000	Ns	31 (1–101)	Routine EEG	<20	4.5%	1.5%

Bhatia et al. (1997) [12]	50	PNES	22.7 (7–51)	STVEEG + saline injection	300	0	60%

PNES: paroxysmal nonepileptic seizure-like symptom.

STVEEG: short term video encephalography.

OVEM: outpatient video EEG monitoring.

E: epilepsy.

P: PNES.

LTVEEG: long term video encephalography.

VS: verbal suggestion.

HV: hyperventilation.

PS: photic stimulation.

ns: not specified.

NC: not confirmed.
